# Case Report: Ninjin'yoeito May Improve Quality of Life After Hospitalization for Acute Illness in Patients With Frailty

**DOI:** 10.3389/fnut.2021.547512

**Published:** 2021-04-14

**Authors:** Masayuki Kashima

**Affiliations:** Department of General Internal Medicine, Japanese Red Cross Kumamoto Hospital, Kumamoto, Japan

**Keywords:** ninjin'yoeito, quality of life, acute illness, frailty, hospitalization

## Abstract

**Introduction:** Frail patients are susceptible to a large number of diseases, and frailty particularly is known to develop after acute illness. No conventional drugs are known to prevent such exacerbation after acute illness. However, traditional Japanese medicine, Kampo, is thought to confer efficacious energy and nutritional supplements and serve to improve malaise after acute illness. Ninjin'yoeito is a representative Kampo medicine for such situation.

**Cases:** We describe three frail patients hospitalized for acute illness who started taking ninjin'yoeito at the time of discharge.

**Case 1:** An 87-year-old man admitted with acute prostatitis complicated by hypertension and chronic obstructive pulmonary disease (COPD). His 36-Item Short Form Health Survey (SF-36) score, which is representative of total quality of life and comprises eight components, showed consistent improvements after 4 and 12 weeks of ninjin'yoeito administration, especially for body pain (BP; scores from 41 to 51 and 100, respectively), social function (SF; 50, 100, 100), and mental health (MH; 75, 75, 90).

**Case 2:** A 65-year-old man admitted with urinary tract infection complicated by primary sclerosing cholangitis and COPD. All SF-36 component scores showed improvement 12 weeks later: physical function (PF; 70–95), role physical (RP; 75–100), BP (72–84), general health (GH; 45–52), vitality (VT; 37.5–75), SF 75–100, role emotional (RE; 75–100), and MH (70–90).

**Case 3:** An 80-year-old man admitted for pneumonia complicated with hypertension. SF-36 score was improved 4 weeks later for RP (68.8–100), BP (52–61), GH (52–72), VT (43.8–62.5), SF (37.5–100), and RE (58.3–91.7).

**Conclusion:** Patients with frailty often have a worsened SF-36 score after discharge following acute illness, but the score may be improved by taking ninjin'yoeito.

## Introduction

Frailty has been attracting increasing attention in today's aging society. It is associated with a need for nursing care, and preventing exacerbation of frailty is crucial. Frail patients are known to be susceptible to a large number of diseases, and frailty particularly is known to develop after acute illness (1, 2). No conventional drugs are known to prevent exacerbation of frailty after acute illness. Currently, only basic interventions such as nutrition therapy and rehabilitation are available to prevent exacerbation of frailty. Traditional Japanese medicine, known as Kampo, is thought to be efficacious as supplements for qi and the blood; qi can be considered to mean vital energy or life energy, and the blood the substance that supplies nutrition. Frailty is considered a state of deficiency of both qi and the blood. The experience of severe disease or trauma exhausts the qi and the blood, leading to frailty.

Drugs that have the effect of supplementing qi and the blood have been used traditionally to prevent or improve the medical condition known as frailty. Ninjin'yoeito, which consists of 12 types of crude drugs and whose basic composition was first described in the thirteenth century, is representative of Kampo medicines and is a supplement for qi and the blood. Some studies have shown that ninjin'yoeito can be effective in improving some frail conditions (3–5). Currently, the Japanese national medical insurance system has approved Kracie ninjin'yoeito extract granules (Kampo Research Laboratory, Kracie Pharma Ltd., Takaoka, Japan) for its efficacy in improving strength after illness. However, few scientific studies have demonstrated this efficacy.

Decreased strength is a part of frailty, yet it is difficult to measure. Attempts have been made to measure it using various indicators, such as quality of life (QOL), nutrition, and muscle strength. The 36-Item Short Form Health Survey (SF-36) is a 36-item, patient-reported survey of patient health. The SF-36 is a measure of health status, and an abbreviated variant of it and one of several established scoring systems for health-related QOL and its reliability and validity have been confirmed. The instrument consists of eight components: physical function (PF), role physical (RP), body pain (BP), general health (GH), vitality (VT), social function (SF), role emotion (RE), and mental health (MH), which are the weighted sums of the questions in their section. Each scale is directly transformed into a 0–100 scale on the assumption that each question carries equal weight. The lower the score means the more disability. The higher the score means the less disability (6–8). Thigh circumference is related to whole body muscle mass and activities of daily living in the elderly (9). Grip strength is known to correlate with overall muscle strength and with mortality (10). Serum albumin level and the Prognostic Nutritional Index [PNI; serum albumin level (mg/dl × 10 + peripheral lymphocyte counts/mm3) × 0.005] are known to correlate with nutritional status and prognosis (11–13).

This report describes three frail patients for whom supplementation with Kracie ninjin'yoeito improved frailty after acute illness, as evaluated using the SF-36, grip strength, serum albumin level, and PNI. The patients were provided with sufficient information and consent regarding the study in writing.

## Cases

The three patients met the criteria for frailty of elderly age, low grip strength (dominant hand grip strength <26 kg in men and <18 kg in women), and low activity (no light exercise once a week) (14).

### Case 1

An 87-year-old man was admitted to our hospital for acute prostatitis. He was treated with antibiotics and hospitalized for 8 days. His condition was complicated by comorbid hypertension, chronic obstructive pulmonary disease (COPD), and mild dementia.

His body length was 168 cm, and at the time of discharge, his body weight was 53.4 kg, body mass index (BMI) was 18.9, serum albumin level was 2.9 g/dl, and PNI was 38.34. He was started on Kracie ninjin'yoeito 7.5 g/day from the day of discharge. Grip strength, femoral circumference, SF-36 score, serum albumin level, and PNI were evaluated at discharge and 4 and 12 weeks later. Body weight increased to 51.8 kg at 4 weeks and 52.6 kg at 12 weeks after discharge. As shown in **Table 2**, grip strength in his dominant right hand increased from 24 kg at discharge to 24.5 kg at 4 weeks after discharge and 27 kg at 12 weeks after discharge. Right thigh circumference increased from 35 cm at discharge to 36 cm and then 38 cm at 4 and 12 weeks, respectively. SF-36 scores at discharge and after 4 and 12 weeks are shown in Table 1: PF 65.0, 75.0, and 50.0; RP 68.8, 68.8, and 56.3; BP 41.0, 51.0, and 100; GH 67.0, 77.0, and 62.0; VT 87.5, 87.5, and 81.3; SF 50.0, 100.0, and 100.0; RE 58.3, 66.7, and 58.3; and MH 75.0, 75.0, and 90.0. Serum albumin level was 4.0 g/dl at both 4 and 12 weeks, and PNI was 49.98 and 50.16, respectively.

**Table 1:** 36-item Short Form Health Survey component scores in 3 frail elderly patients who received ninjin'yoeito from the time of discharge after acute illness.

	**Case**	**Discharge**	**4 weeks**	**12 weeks**
PF	1	65	75	50
	2	70	–	95
	3	70	40	–
	Average	68.3	57.5	72.5
RP	1	68.8	68.8	56.3
	2	75	–	100
	3	68.8	100	–
	Average	70.9	84.4	78.2
BP	1	41	51	100
	2	72	–	84
	3	52	61	–
	Average	55	56	92
GH	1	67	77	62
	2	45	–	52
	3	52	72	–
	Average	54.7	74.5	57
VT	1	87.5	87.5	81.3
	2	37.5	–	75
	3	43.8	62.5	–
	Average	56.3	75	78.2
SF	1	50	100	100
	2	75	–	100
	3	37.5	100	–
	Average	54.2	100	100
RE	1	58.3	66.7	58.3
	2	75	–	100
	3	58.3	91.7	–
	Average	63.9	79.2	79.2
MH	1	75	75	100
	2	70	–	90
	3	95	85	–
	Average	80	80	95

*PF, physical function; RP, role physical; BP, body pain; GH, general health; VT, vitality SF, social function; RE, role emotion; MH, mental health*.

### Case 2

A 65-year-old man was admitted to our hospital for urinary tract infection. He was treated with antibiotics and hospitalized for 7 days. His condition was complicated by primary sclerosing cholangitis, COPD, and diabetes mellitus.

His body length was 173.4 cm, body weight was 47.8 kg, BMI was 15.9, serum albumin level was 3.0 g/dl, and PNI was 34.94 at discharge. He was started on Kracie ninjin'yoeito 7.5 g/day from the day of discharge. Grip strength, femoral circumference, SF-36 score, serum albumin level, and PNI were evaluated at discharge and 12 weeks later. Body weight increased to 50.0 kg at 12 weeks after discharge. As shown in Table 2, grip strength in his dominant right hand improved from 28 kg at discharge to 31 kg at 12 weeks. Right thigh circumference increased from 27 to 33 cm. SF-36 scores at discharge and 12 weeks are shown in Table 1: PF 70.0 and 95.0; RP 75.0 and 100.0, BP 72.0 and 84.0, GH 45.0 and 52.0, VT 37.5 and 75.0, SF 75.0 and 100.0, RE 75.0 and 100.0, and MH 70.0 and 90.0. Serum albumin level was 3.6 g/dl, and PNI was 40.94 at 12 weeks after discharge.

**Table 2:** PNI in our 3 elderly patients taking ninjin'yoeito after discharge.

		**Case 1**	**Case 2**	**Case 3**	**Average**	**Percent average change**
Body mass index		18.9	15.9	25.5	20.1	–
Grip strength (kg)	Discharge	24	28	17	23.0	–
	4 weeks	24.5	–	20	22.3	+9.86%
	12 weeks	27	31	–	29.0	+11.60%
Femoral circumference(cm)	Discharge	35	27	44		–
	4 weeks	36	–	45.7		+3.35%
	12 weeks	38	33	–		+15.39%
Serum albumin (mg/dL)	Discharge	2.9	3.0	2.6		–
	4 weeks	4.0	–	4.3		+51.65%
	12 weeks	4.0	3.6	–		+38.97%
PNI	Discharge	38.34	34.94	32.8		–
	4 weeks	49.98	–	54.95		+48.94%
	12 weeks	50.16	40.94	–		+23.99%

*Grip strength: measured in dominant hand*.

*Femoral circumference: measured in the leg of the dominant hand side*.

*PNI: Prognostic Nutritional Index; serum albumin level (mg/dL) × 10+ peripheral lymphocyte counts (/mm^3^) × 0.005*.

### Case 3

An 80-year-old man was admitted to our hospital for community-acquired pneumonia. He was treated with antibiotics and hospitalized for 8 days. His condition was complicated by diabetes mellitus and hypertension.

His body length was 158 cm, body weight was 63.7 kg, BMI was 25.5, serum albumin level was 2.6 g/dl, and PNI was 32.8 at discharge. He was started on Kracie ninjin'yoeito 7.5 g/day from the day of discharge. His grip strength, femoral circumference, SF-36 score, serum albumin level, and PNI were evaluated at discharge and 4 weeks later. Body weight increased to 65 kg at 4 weeks after discharge. As shown in Table 2, grip strength in his dominant right hand increased from 17 kg at discharge to 20 kg at 4 weeks after discharge. Right thigh circumference increased from 44 cm at discharge to 45.7 cm at 4 weeks after discharge. Corresponding SF-36 scores are shown in Table 1: PF 70.0 and 40.0, RP 68.8 and 100.0, BP 52.0 and 61.0, GH 52.0 and 72.0, VT 43.8 and 62.5, SF 37.5 and 100.0, RE 58.3 and 91.7, and MH 95.0 and 85.0. Serum albumin level was 4.3 g/dl, and PNI was 54.95 at 4 weeks after discharge.

**Figure 1:**
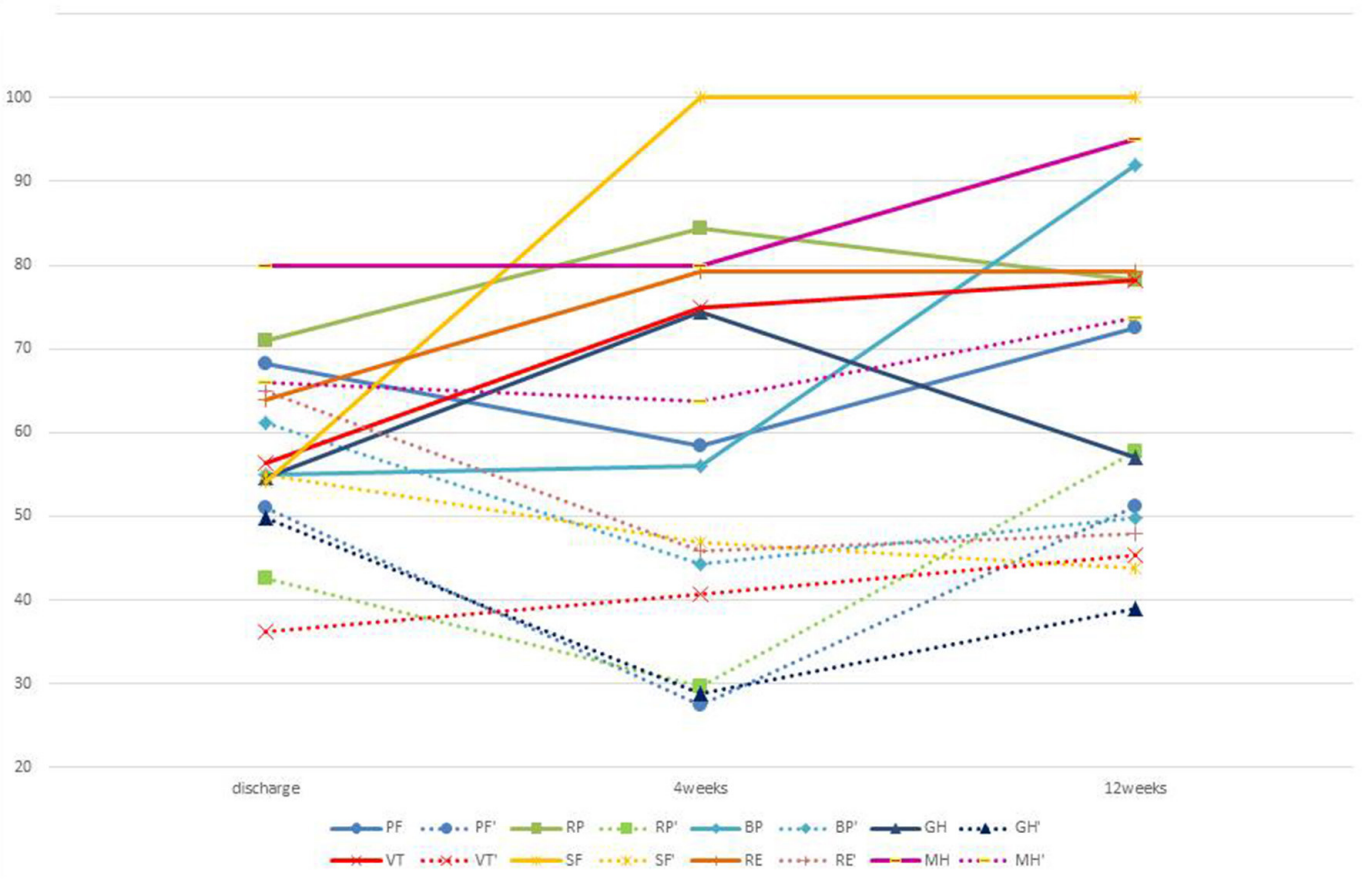
Average transition of SF36 with and without ninjin'yoeito. PF, RP, BP, GH, VT, SF, RE, MH: average value of SF-36 of three people who received ninjin'yoeito. RF', RP', BP', GH', VT', SF', RE', and MH': average value of SF-36 of five people who did not receive ninjin'yoeito.

Overall, the average changes in the component SF-36 scores from discharge in these three cases were as follows: PF −10.00 at 4 weeks and +5.00 at 12 weeks after discharge, RP +15.60 and +6.25, BP +9.50 and +35.5, GH +15.00 and +1.00, VT +9.35 and +15.65, SF +56.25 and 37.50, RE +20.90 and +12.50, and MH −5.00 and +22.50 (Figure 1) (1, 2).

**Figure 2:**
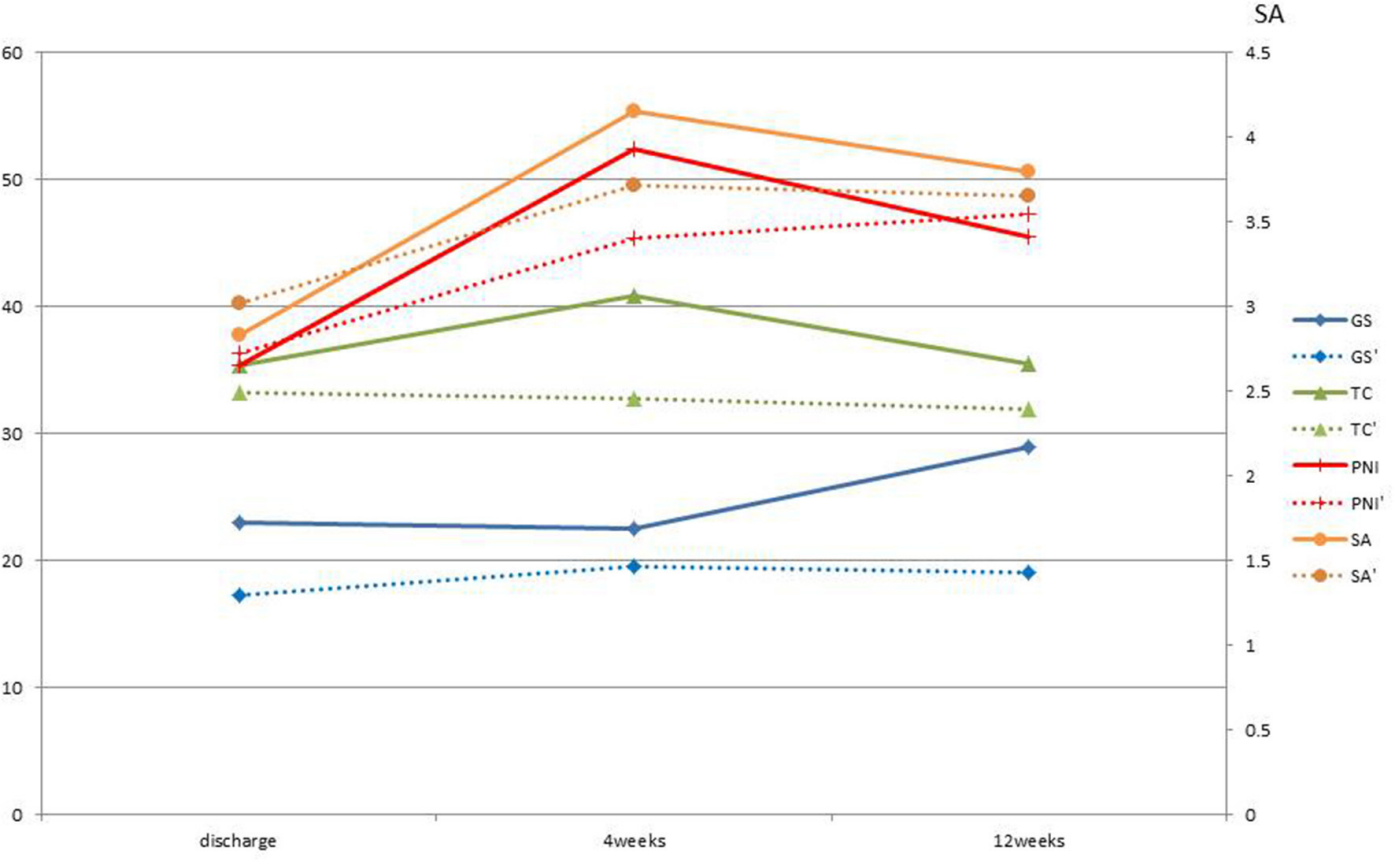
Average change from discharge of each index with and without ninjin'yoeito. GS, Grip strength; kg; TC, Thigh circumference: cm; SA, Serum albumin; mg/dl; PNI, Prognostic Nutritional Index are the average change from discharge of the three patients who received ninjin'yoeito. GS', Grip strength; TC', Thigh circumference; SA', Serum albumin; PNI', Prognostic Nutritional Index are the average change from discharge of five patients who did not received ninjin'yoeito.

The average change in grip strength at discharge was +1.75 kg at 4 weeks and +3 kg at 12 weeks after discharge. The corresponding change in femoral circumference was +1.35 and 4.5 cm, in serum albumin level was +1.4 and +0.85 mg/dl, and in PNI was +16.89 and +8.88 (Figure 2) (1, 2).

## Discussion

This report has described our experience treating three frail patients with ninjin'yoeito after being discharged after acute illness and the beneficial effects it showed. We also have experience with five other frail patients (age, 66–90 years) who were discharged with acute illness who were not placed on ninjin'yoeito and were followed up for measurements of SF-36 score, grip strength, serum albumin, and PNI at discharge and for prognostic indices on an outpatient basis. The average change in SF-36 scores for these five patients from discharge to 4 and 12 weeks after discharge, respectively, were as follows: −13.75 and +5.00 for PF, −4.7 and +18.75 for RP, −29.25 and −8.75 for BP, −20.5 and −9.5 for GH, +4.67 and +14.05 for VT, −12.50 and −12.50 for SF, −41.70 and −8.32 for RE, and +1.25 and +10.00 for MH (Figure 1) (1, 2). The average grip strength was 13.30 kg at discharge, with the average change in grip strength +2.50 kg at 4 weeks and +2.16 kg at 12 weeks. The corresponding average change in femoral circumference was −0.70 and −0.43 cm, in serum albumin level from discharge was +0.70 and +0.82 mg/dl, and the average change in PNI was +9.07 and +12.51 (Figure 2) (1, 2). Comparing all three patients who received ninjin'yoeito treatment with the five patients who did not receive it showed that the SF-36 scores for GH, BP, SF, and RE seem to have improved markedly, and scores for RP and MH showed a slight improvement in the three patients who received ninjin'yoeito. Scores for PF and VT were almost the same in the two groups. MH, SF, and RE reflect psychosocial status. Pain is known to be related to psychological factors. Therefore, BP may have improved due to the psychological effect of ninjin'yoeito. In both groups, PH tended to decline after discharge (Figure 1) (1, 2). It is, however, possible that their scores at discharge were higher than their actual scores because baseline scores were measured during hospitalization, when exercise was restricted compared with daily life, and it was not possible to accurately define their level of activity. Also, activity may have decreased due to progression of underlying disease. Hochuekkito is a representative Kampo supplement for qi and is reported to improve SF-36 score (15). Ninjin'yoeito contains crude drugs such as Astragali radix, Ginseng, *Atractylodis* rhizome, and licorice, which are also components of hochuekkito and are thought to be qi supplements, so it is expected that SF-36 can be improved with ninjin'yoeito. Traditionally, ninjin'yoeito is considered to have an effect on the heart, which is the center of mental function that is addressed in Kampo medicine and is thought to be more likely to produce a mental effect.

Cases 1 and 2 have COPD. Hochuekkito is reported to improve total CAT (COPD assessment test) score and dyspnea, fatigue in COPD patients (16). As mentioned above, because ninjin'yoeito contains similar herbs to hochuekkito, ninjin'yoeito may improve dyspnea and fatigue as well. This may have contributed to the improvement of SF-36, especially VT in Cases 1 and 2. In addition, ninjin'yoeito is a Kampo supplement for not only qi but also the blood. Ninjin'yoeito may contribute to the improvement of nutritional status in COPD patients

In terms of grip strength, there was no significant change between the group treated with ninjin'yoeito and the group that was not treated with it. However, half of those not treated with ninjin'yoeito showed no change, whereas all the three subjects in the ninjin'yoeito-treated group showed improvement in grip strength. A previous randomized controlled trial reported an oral ninjin'yoeito administration group also showed improved grip strength (Figure 2) (1, 2, 5).

Regarding serum albumin and PNI, no significant difference was observed between the group that received ninjin'yoeito and the group that did not. However, Case 2 was complicated with primary sclerosing cholangitis. It might be possible that the poor improvement in serum albumin after 12 weeks was due to reduced albumin production capacity. PNI showed an improvement rate similar to that of albumin in both groups (Figure 2) (1, 2).

There are some limitations of this open-label pilot case report. First, the placebo effect was not taken into account especially in terms of the psychological effect because this was not a placebo-controlled trial. Second, this was not a randomized controlled trial, so the conditions of the two groups, in terms of treatment with ninjin'yoeito, were not the same. Thus, no test for significant difference could be applied for comparison between the two groups. Large randomized controlled trials are needed.

## Data Availability Statement

The raw data supporting the conclusions of this article will be made available by the authors, without undue reservation, to any qualified researcher.

## Ethics Statement

Written informed consent was obtained from the individual(s) for the publication of any potentially identifiable images or data included in this article.

## Author Contributions

The author confirms being the sole contributor of this work and has approved it for publication.

## Conflict of Interest

The author declares that this study received funding from Kracie Pharmaceutical Co., Ltd. The funder was not involved in the study design, collection, analysis, interpretation of data, the writing of this article or the decision to submit it for publication.
