# Supplementary material for: Case Report: Ninjin'yoeito May Improve Quality of Life After Hospitalization for Acute Illness in Patients With Frailty
Source: Front Nutr. 2021 Apr 14;8:547512. doi: 10.3389/fnut.2021.547512 (PMC8079971; doi:10.3389/fnut.2021.547512)
Supplement: Supplementary file 2 [file Table_2.DOCX]

|  | PF | RP | BP | GH | VT | SF | RE | MH | 合計 |
| --- | --- | --- | --- | --- | --- | --- | --- | --- | --- |
| 0 | 60.0 | 68.8 | 100.0 | 75.0 | 68.8 | 100.0 | 100.0 | 70.0 | 642.5 |
| 4 | 35.0 | 25.0 | 52.0 | 30.0 | 50.0 | 62.5 | 50.0 | 60.0 | 364.5 |
| 12 | 70.0 | 50.0 | 52.0 | 37.0 | 43.8 | 50.0 | 66.7 | 75.0 | 444.4 |
|  |  |  |  |  |  |  |  |  |  |
| ０ | 20.0 | 12.5 | 100.0 | 20.0 | 0.0 | 50.0 | 0.0 | 60.0 | 262.5 |
| 4 | 15.0 | 18.8 | 32.0 | 20.0 | 43.8 | 25.0 | 33.3 | 65.0 | 252.8 |
| 12 | 40.0 | 100.0 | 54.0 | 20.0 | 37.5 | 12.5 | 25.0 | 80.0 | 369.0 |
|  |  |  |  |  |  |  |  |  |  |
| 0 | 90.0 | 75.0 | 12.0 | 52.0 | 37.5 | 37.5 | 100.0 | 70.0 | 474.0 |
| 12 | 95.0 | 81.3 | 62.0 | 57.0 | 50.0 | 75.0 | 100.0 | 85.0 | 605.3 |
|  |  |  |  |  |  |  |  |  |  |
| 0 | 70.0 | 56.3 | 72.0 | 55.0 | 56.3 | 50.0 | 100.0 | 75.0 | 534.5 |
| 4 | 60.0 | 75.0 | 61.0 | 30.0 | 37.5 | 75.0 | 100.0 | 80.0 | 518.5 |
|  |  |  |  |  |  |  |  |  |  |
| 0 | 15.0 | 0.0 | 22.0 | 47.0 | 18.8 | 37.5 | 25.0 | 55.0 | 220.3 |
| 4 | 0.0 | 0.0 | 32.0 | 35.0 | 31.3 | 25.0 | 0.0 | 50.0 | 173.3 |
| 12 | 0.0 | 0.0 | 31.0 | 42.0 | 50.0 | 37.5 | 0.0 | 55.0 | 215.5 |
